# Noninvasive detection of twin zygosity using genome‐wide linkage disequilibrium information

**DOI:** 10.1002/ctm2.70130

**Published:** 2024-12-19

**Authors:** Lingrong Kong, Yingjun Yang, Chao Yuan, Xing Wei, Xinyao Zhou, Jia Zhou, Ya Xing, Gang Zou, Qianqian Sun, Luyao Cai, Qiufeng Liang, Yao Zhang, Hongkun Wang, Zesi Liu, Di Wu, Luming Sun

**Affiliations:** ^1^ Department of Fetal Medicine & Prenatal Diagnosis Center Shanghai Key Laboratory of Maternal Fetal Medicine Shanghai First Maternity and Infant Hospital School of Medicine Tongji University Shanghai China; ^2^ Research and Development Department Celula (China) Medical Technology Co., Ltd. Chengdu China; ^3^ Department of Obstetrics, Center of Fetal Medicine & intrauterine pediatrics Xinhua Hospital, Shanghai Jiao Tong University School of Medicine Shanghai China

## Abstract

Dear Editor, in this study, we propose a novel linkage disequilibrium information‐based noninvasive zygosity (LDNZ) assessment method in twin pregnancies. It combines fetus‐specific allele frequency analysis with LD block to reduce the number of required single nucleotide polymorphism markers and experiment costs. LDNZ method offers a noninvasive, accurate, and cost‐effective solution for zygosity assessment, addressing the need for precise obstetric care in twin pregnancies.

1

Dear Editor,

In this study, we propose a novel linkage disequilibrium information‐based noninvasive zygosity (LDNZ) assessment method in twin pregnancies. It combines fetus‐specific allele frequency analysis with LD block to reduce the number of required single nucleotide polymorphism (SNP) markers and experiment costs. LDNZ method offers a noninvasive, accurate, and cost‐effective solution for zygosity assessment, addressing the need for precise obstetric care in twin pregnancies.

The proportion of multiple pregnancies has been increasing due to the widespread use of ovulation drugs in assisted reproductive technology.^1^ Determining zygosity is essential for the prenatal diagnosis of single‐gene disorders in twin pregnancies. In the first trimester, having prior knowledge of twin zygosity allows obstetric care providers to determine whether invasive prenatal diagnosis is necessary for each fetus. In the second trimester, amniocentesis results showing identical genotypes for both fetuses in DCDA twins most likely indicate monozygotic (MZ) twins. However, this could also result from the repeated sampling of the same fetus. SNP‐based noninvasive zygosity assessment can assist clinicians in excluding the possibility of re‐sampling. An accurate zygosity assessment is essential for obtaining reliable results in noninvasive prenatal testing (NIPT) for aneuploidy in twin pregnancies.^2^ The zygosity affects the prior risk of chromosomal abnormalities differently in dizygotic (DZ) twins, as the aneuploid incidence is independent for each fetus.^3^

SNP‐based noninvasive zygosity assessment has been proven to be highly clinically applicable. This methodology incorporated 207 target LD blocks with only 1035 SNPs (Figure 1A). SNPs were classified into three categories based on the mother and alleged father genotypes (Figure 1B). For MZ twins, category 2 SNPs have the same binomial distributions as Category 1 and Category 3 SNPs (See Table S1–S6 and Figure S1–S2 in Supporting Information). However, for DZ twins Category 2 SNPs have additional binomial distribution (Figure 2A,B).

**FIGURE 1:**
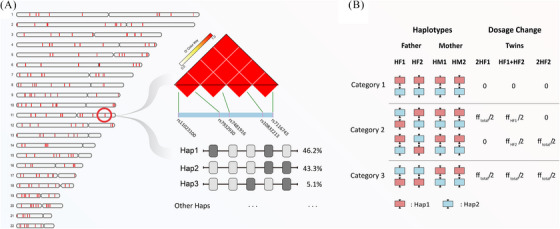
Schematic representation of linkage disequilibrium (LD)‐blocks on autosomal chromosomes. (A) The distribution of 1–22 autosomal chromosomes comprising 207 blocks, with red areas indicating block regions. Display linkage disequilibrium analysis plots for five single nucleotide polymorphism (SNP) markers within the chr11: 14160504–14202168 region, along with the probabilities of the two main haplotypes (hap1 and hap2). (B) The classification principle for SNP sites is based on maternal and corresponding paternal samples. Red squares represent hap1, while blue squares represent hap2.

**FIGURE 2:**
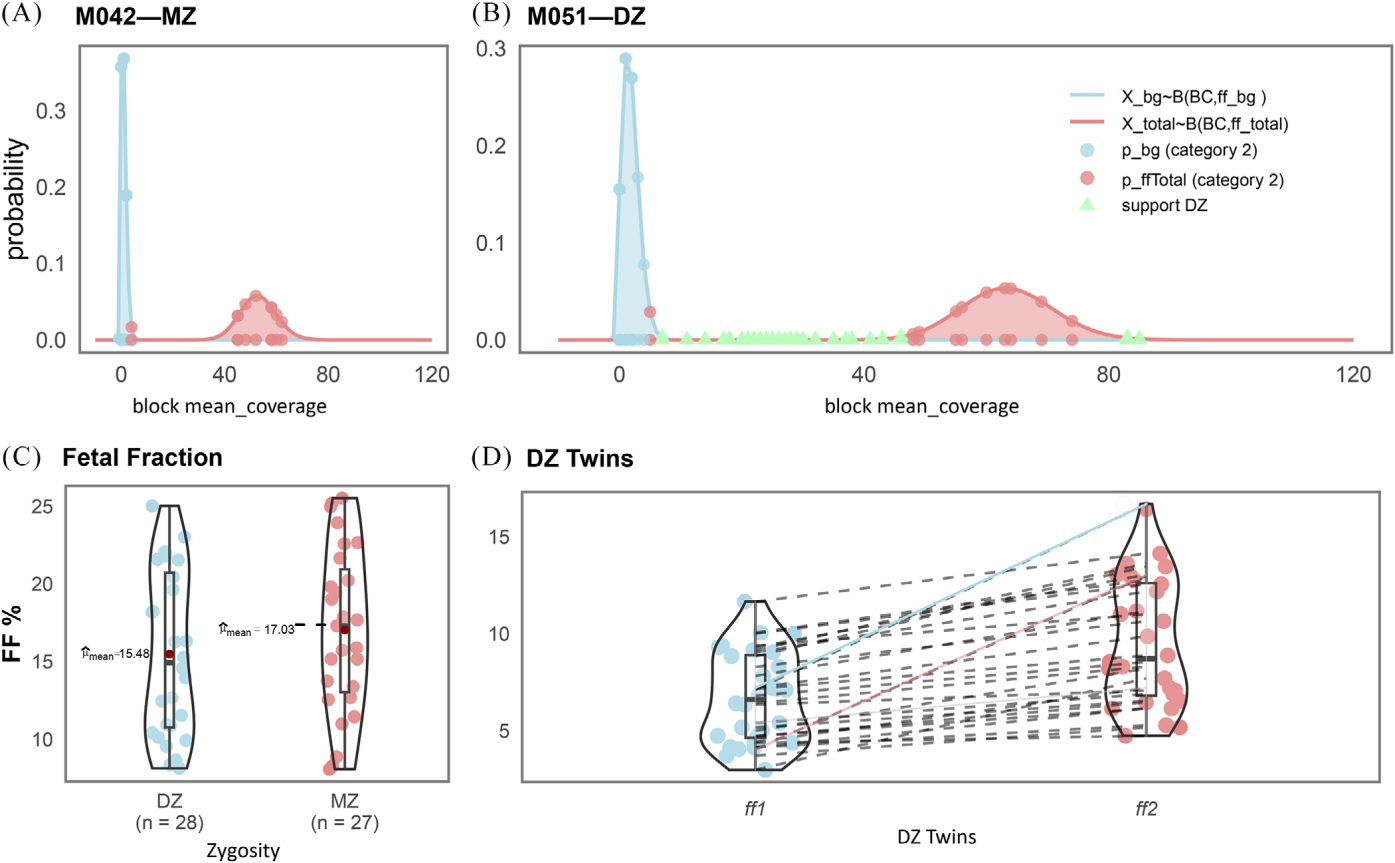
Linkage disequilibrium information‐based noninvasive zygosity (LDNZ) for zygosity assessmnet and fetal fraction evaluation (A) Binomial distribution for the M042 twin. None of the Category 2 blocks met the conditions of *p*_bg_ ≤ .05 and *p*_Total_ ≤ .05. M042 was identified as the monozygotic twin. (B) Binomial distribution for M051 twin. 33 Category 2 blocks met the conditions of *p*_bg_ ≤ .05 and *p*_Total_ ≤ .05. M051 was identified as the dizygotic twin. (C) The distribution of total fetal fraction for 27 MZ twins and 28 DC twins. (D) The distribution of individual fetal fraction for 28 DC twins (*ff*1 < *ff*2). The difference between individual fetal fractions in M034 and M043 twins shows with multiples of 3.4‐fold (red highlight line) and 2.8‐fold (blue highlight line).

Fifty‐five twin pregnancies were recruited for twin zygosity assessment, with results showing 100% consistency (55/55) with short tandem repeat (STR) typing. LDNZ zygosity assessment revealed 28 cases of DZ twins and 27 cases of MZ twins (Table 1). The average informative block for DZ twins was 28, and the average for MZ twins was 0. The total fetal fraction of MZ twins ranged from 8.56% to 25.52%, with an average of 17.03%. Dizygotic twins exhibited a total fetal fraction ranging from 8.12% to 25.02%, with an average total fetal fraction of 15.48% (Figure 2C,D). Additionally, we compared the LDNZ method and the classical SNP‐based method for zygosity assessments (Figure 3). The sensitivity and specificity of the LDNZ method were 1, while the SNP‐based method misdiagnosed seven MZ twins as DZ twins (Table S5).

**TABLE 1:** Detailed information for the analysis of fetus‐specific linkage disequilibrium‐single nucleotide polymorphism (LD‐SNP) alleles in cell‐free DNA (cfDNA) from 55 twin pregnancies.

Sample ID	Category 1 Block	Category 2 Block	Category 3 Block	Informative Block (n)	ff_total_ (%)	ff_1_ (%)	ff_2_ (%)	LDNZ Zygote	STR Zygote
**M001**	20	53	26	0	11.38	NA	NA	MZ	MZ
**M002**	20	55	35	4	9.32	4.03	4.92	DZ	DZ
**M003**	28	49	30	9	11.62	4.37	5.84	DZ	DZ
**M004**	25	53	28	12	22.04	7.51	11.67	DZ	DZ
**M005**	20	55	29	0	18.78	NA	NA	MZ	MZ
**M006**	28	40	29	0	14.54	NA	NA	MZ	MZ
**M007**	27	49	30	0	23.20	NA	NA	MZ	MZ
**M008**	29	51	30	12	9.72	3.55	4.78	DZ	DZ
**M009**	32	54	20	0	25.78	NA	NA	MZ	MZ
**M010**	25	49	29	20	18.48	8.47	10.31	DZ	DZ
**M011**	26	54	23	12	9.54	2.48	4.43	DZ	DZ
**M012**	33	59	20	0	21.50	NA	NA	MZ	MZ
**M013**	20	53	23	4	11.52	4.7	5.93	DZ	DZ
**M014**	35	43	18	17	18.48	5.59	9.88	DZ	DZ
**M015**	22	51	31	0	10.6	NA	NA	MZ	MZ
**M016**	26	43	33	0	25.00	NA	NA	MZ	MZ
**M017**	23	58	20	24	17.48	7.66	10.19	DZ	DZ
**M018**	24	53	23	0	13.54	NA	NA	MZ	MZ
**M019**	28	52	27	16	21.76	8.77	12.42	DZ	DZ
**M020**	25	60	23	25	23.08	9.15	13.17	DZ	DZ
**M021**	25	54	23	24	10.12	3.38	5.49	DZ	DZ
**M022**	25	50	29	14	13.58	4.94	7.29	DZ	DZ
**M023**	31	49	25	0	13.18	NA	NA	MZ	MZ
**M024**	29	59	25	0	19.42	NA	NA	MZ	MZ
**M025**	27	56	23	35	20.00	9.17	11.60	DZ	DZ
**M026**	25	56	26	24	19.36	8.65	11.89	DZ	DZ
**M027**	24	41	32	25	18.26	8.79	11.44	DZ	DZ
**M028**	34	58	26	10	7.82	2.5	3.58	DZ	DZ
**M029**	32	56	23	0	16.98	NA	NA	MZ	MZ
**M030**	31	43	24	18	10.20	4.37	5.85	DZ	DZ
**M031**	26	54	40	16	13.38	4.57	7.55	DZ	DZ
**M032**	24	51	24	16	10.22	4.51	5.91	DZ	DZ
**M033**	29	40	26	0	18.96	NA	NA	MZ	MZ
M034^*^	28	50	31	17	15.38	4.73	9.69	DZ	DZ
**M035**	17	53	22	0	14.60	NA	NA	MZ	MZ
**M036**	31	50	36	0	20.30	NA	NA	MZ.	MZ
**M037**	25	56	17	0	8.70	NA	NA	MZ	MZ
**M038**	21	51	22	0	12.50	NA	NA	MZ	MZ
**M039**	27	53	25	0	10.86	NA	NA	MZ	MZ
**M040**	35	52	30	0	17.06	NA	NA	MZ	MZ
**M041**	28	52	30	0	7.12	NA	NA	MZ	MZ
**M042**	25	47	30	0	18.34	NA	NA	MZ	MZ
M043^*^	35	61	24	22	9.18	2.47	6.22	DZ	DZ
**M044**	34	52	23	0	24.30	NA	NA	MZ	MZ
**M045**	24	52	27	24	21.54	8.30	13.06	DZ	DZ
**M046**	25	59	23	0	11.64	NA	NA	MZ	MZ
**M047**	29	47	29	0	16.72	NA	NA	MZ	MZ
**M048**	32	55	23	0	24.60	NA	NA	MZ	MZ
**M049**	21	52	25	0	20.28	NA	NA	MZ	MZ
**M050**	29	52	24	16	7.62	3.34	4.15	DZ	DZ
**M051**	23	63	26	33	22.22	8.73	13.34	DZ	DZ
**M052**	30	47	31	27	13.22	5.34	7.22	DZ	DZ
**M053**	26	52	22	0	14.44	NA	NA	MZ	MZ
**M054**	23	53	25	18	16.18	6.50	9.68	DZ	DZ
**M055**	24	51	22	18	11.32	5.98	7.47	DZ	DZ

**FIGURE 3:**
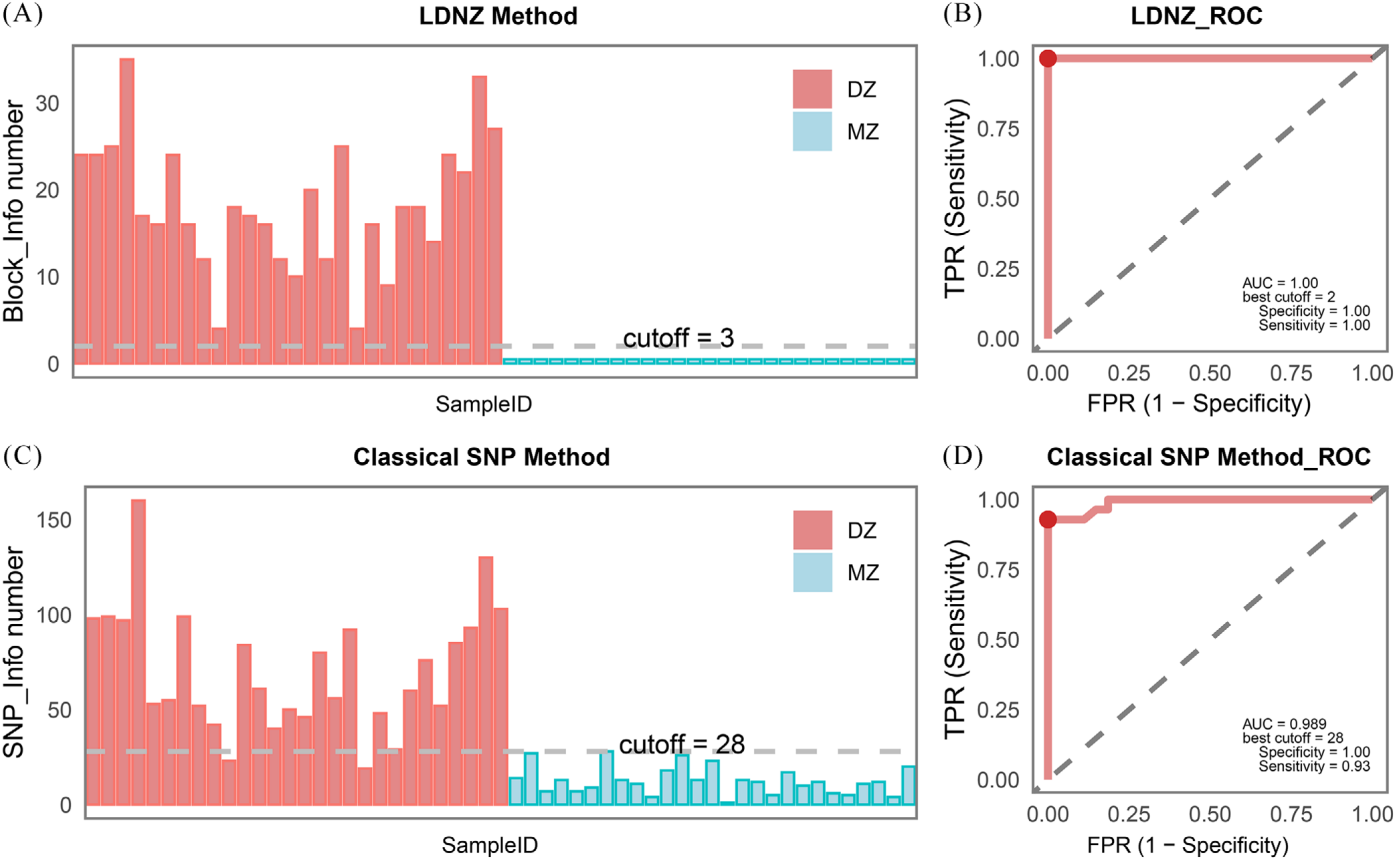
Histograms and receiver operating characteristic (ROC) analysis for linkage disequilibrium information‐based noninvasive zygosity (LDNZ) method and classical single nucleotide polymorphism (SNP)‐based method (A) Histogram showing the distribution of the number of informative blocks identified by the LDNZ analysis. (B) ROC curve analysis for the number of informative blocks identified by the LDNZ method. At the optimal model prediction performance, the best cutoff value is 2, with specificity, sensitivity, and area under the curve (AUC) equal to 1. (C) Histogram showing the distribution of the number of informative loci identified by the SNP analysis. (D) ROC curve analysis for the number of informative SNPs identified by the SNP method. At the optimal model prediction performance, the best cutoff value is 28, with a specificity of 1.00, a sensitivity of 0.93, and an AUC of 0.989.

Various studies have confirmed the potential of using biallelic SNP loci for noninvasive zygosity determination. For instance, Leung et al. used 900 000 SNP loci on noninvasive prenatal zygosity, necessitating 431 million reads.^4^ Similarly, Zheng et al. used over 4524 SNP loci, requiring 11.5 million reads.^5^ While Hedriana et al. utilized over 13 000 SNP loci, necessitating 28 million reads.^6^ Even the latest research in 2023, despite optimizing algorithms, required over 2800 SNP loci.^7^ The classical SNP‐based approach demands a substantial number of SNPs, leading to increased costs and more complex data analysis. In contrast, our experiment utilized only 1035 SNP loci, merely requiring 1.5 million reads. LDNZ offers significant savings and reduces experimental costs per sample to less than $15 (Table S6). Compared to classical SNP‐based zygosity detection methods, LDNZ offers greater affordability and simplicity, making it a more accessible option.

Unlike SNP‐based noninvasive prenatal zygosity detection methods, some studies have proposed alternative approaches using other markers. Dziennik et al. used STRs and deletion/insertion polymorphism (DIP)‐STR compounds to assess zygosity by identifying fetal alleles in maternal cell‐free DNA.^8^ Including 28 twin pregnancies, the sensitivity of this method was 80%, with a specificity of 81.25%. A limitation of this assay is the potential for false positive results (incorrect identification of DZ pregnancy) due to stutters generated during STR or DIP‐STR amplification. Furthermore, Bai et al. employed a method utilizing 60 microhaplotypes (closely linked SNP loci < 200 bp) to assess zygosity and fetal fraction by examining the complementary genomic information of the two fetuses.^9^ However, a particular limitation of microhaplotypes assays is the limited number of informative loci in some studied cases, which are also the threshold for distinguishing MZ and DZ twins. STR/(DIP)‐STR compounds and microhaplotypes are not included in the analysis of twin aneuploidy, as they cannot be concurrently assessed with NIPT for twin aneuploidy.

The LDNZ method could enhance accuracy in zygosity assessment. The same allele frequency of SNP loci in the LD block could balance sequencing error. The mean‐variance of linked SNP allele frequency is extremely low, averaging 0.0099. In contrast, individual unlinked SNP loci are more susceptible to experimental sampling error and sequencing bias, leading to more significant fluctuations in fetal allele frequencies. The mean‐variance of the classical SNP‐based method reaches 0.0395, which is 3.9‐fold higher than that of the LDNZ method. In our previous study, LDNZ combined with relative haplotype dosage analysis (RHDO) has been utilized for noninvasive prenatal diagnosis for recessive single‐gene disorders and zygosity assessment in twin pregnancies.^10^

Besides acting as a guide or internal control for prenatal genetic tests, the individual fetal fraction in DZ twins could also help obstetricians track the growth of each fetus. Previous research suggests that fetuses in the DZ twin contribute different amounts of fetal fraction (up to a 2‐fold difference) to maternal circulation.^4^ Recent research has shown that a low fetal fraction in the population without obesity was associated with higher systolic blood pressure during pregnancy. In this study, the individual fetal fraction (*ff_1_* and *ff_2_*) of DZ twins M034 and M043 differed significantly, with multiples of 3.4‐ and 2.8‐fold (Table 1 and Figure 2D), respectively. Both M034 and M043 exhibited preeclampsia in the third trimester. In the future, we prepare to investigate the correlation between the degree of differences in the individual fetal fraction of twins and preeclampsia.

In conclusion, the LDNZ method is introduced for noninvasive zygosity assessment, leveraging linkage disequilibrium information. This approach significantly decreases the required number of SNPs and reduces detection costs. This novel method represents an advancement in the field of noninvasive prenatal zygosity screening, offering improved care for twin pregnancies.

## AUTHOR CONTRIBUTIONS

Kong Lingrong: Conceptualization; Methodology; Validation; Formal analysis; Investigation; Resources; Data Curation; Writing—original draft; Visualization. Yang Yingjun: Conceptualization; Methodology; Resources. Yuan Chao: Methodology; Software; Data Curation. Gang Zou: Resources. Xinyao Zhou: Resources. Jia Zhou: Resources. Xing Ya: Resources. Wei Xing: Resources. Qianqian Sun: Resources. Luyao Cai: Resources. Qiufeng Liang: Resources. Yao Zhang: Resources. Hongkun Wang: Resources. Zesi Liu: Resources. Wu Di: Methodology; Writing—review & editing; Supervision. Luming Sun: Writing—Review & Editing; Supervision; Project administration and funding acquisition.

## CONFLICT OF INTEREST STATEMENT

Chao Yuan and Di Wu are employed by Celula (China) Medical Technology Co., Ltd. The remaining authors declare no conflict of interest.

## FUNDING INFORMATION

The research was funded by the National Key Research and Development Program of China (Nos. 2022YFC2704700 and 2018YFC1002900), the National Natural Science Foundation of China (82071656) and the Shanghai Municipal Science and Technology Commission (Nos. 21Y11907500 and 23DZ2303400).

## ETHICS STATEMENT

This study protocol was approved by the institutional review board of Shanghai First Maternity and Infant Hospital (GKLW2019‐72 and GKLW2019‐52).

## PATIENT CONSENT STATEMENT

Informed consent was obtained from all participants.

## Supporting information



Supporting information

Supporting information

Supporting information

Supporting information

## Data Availability

The datasets for this article are not publicly available due to concerns regarding participant/patient anonymity. Requests to access the datasets should be directed to the corresponding authors.
