# Supplementary figures and images for: Noninvasive detection of twin zygosity using genome‐wide linkage disequilibrium information
Source: Clin Transl Med. 2024 Dec 19;14(12):e70130. doi: 10.1002/ctm2.70130 (PMC11659192; doi:10.1002/ctm2.70130)

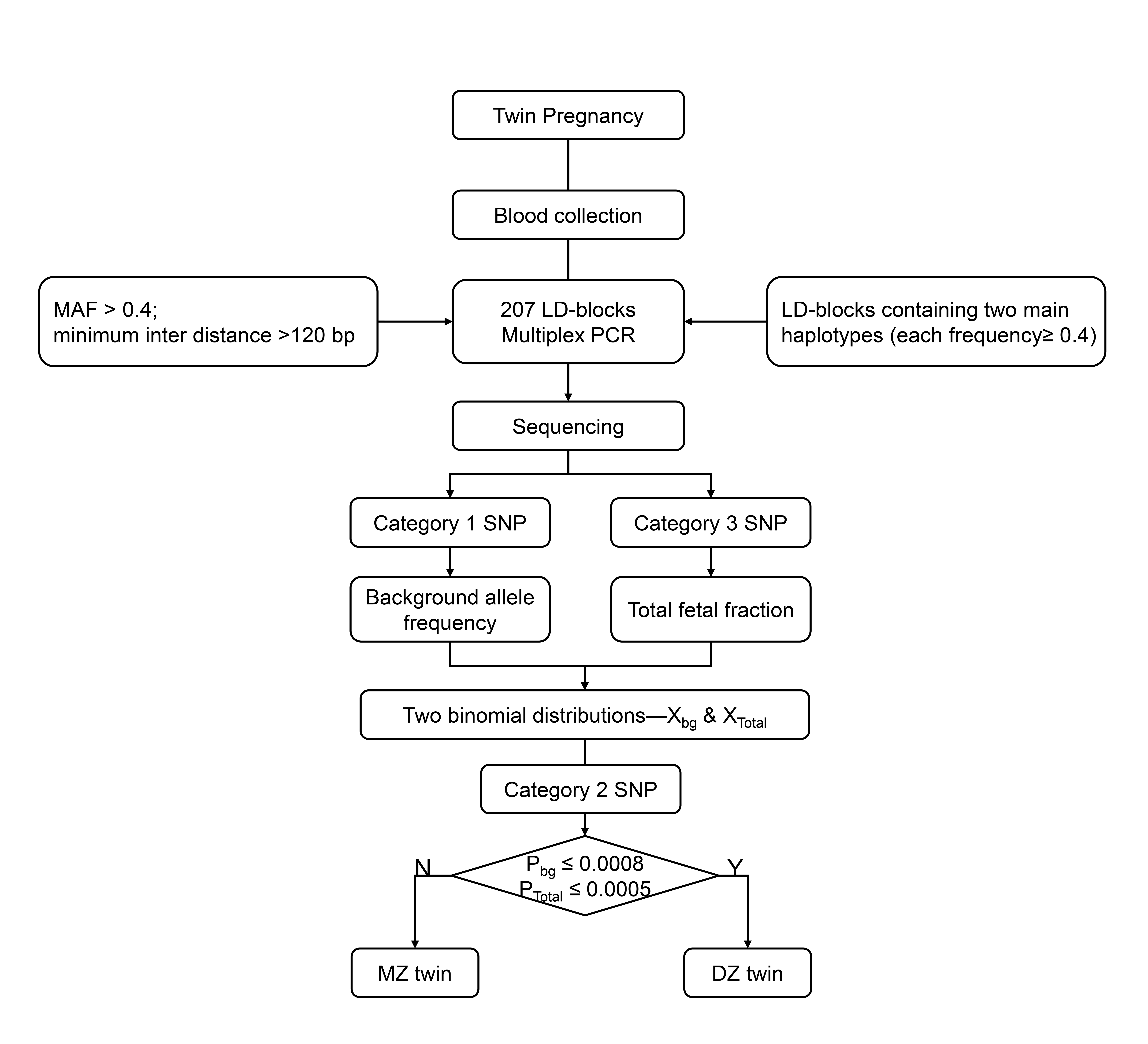

Supplement: Supplementary file 1 — Supporting information [file CTM2-14-e70130-s001.tif]

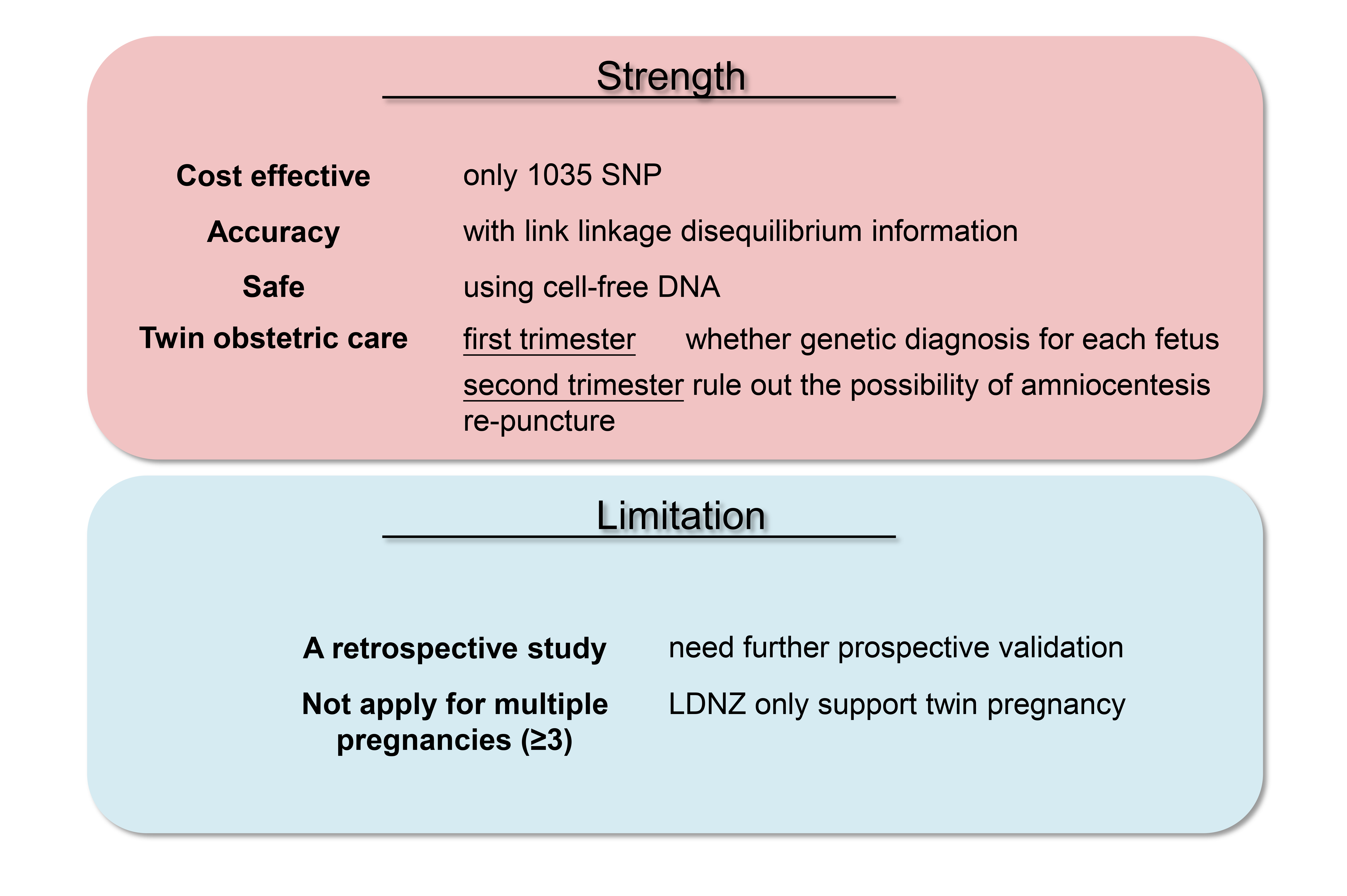

Supplement: Supplementary file 2 — Supporting information [file CTM2-14-e70130-s002.tif]
