# Supplementary material for: Noninvasive detection of twin zygosity using genome‐wide linkage disequilibrium information
Source: Clin Transl Med. 2024 Dec 19;14(12):e70130. doi: 10.1002/ctm2.70130 (PMC11659192; doi:10.1002/ctm2.70130)
Supplement: Supplementary file 4 — Supporting information [file CTM2-14-e70130-s004.docx]

Letter to the Journal

Manuscript ID CTM2-2024-08-2835

**Noninvasive Detection of Twin Zygosity Using Genome-wide Linkage Disequilibrium Information**

**List of Supplementary Information**

- **Materials and Methods**
- **Result**

**Materials and Methods**

**Sample Collection and Preparation**

Samples were collected between January 2020 and December 2021 in the Shanghai First Maternity and Infant Hospital. We recruited 55 twin pregnancies with informed consent obtained from all participants. Pregnant women should undergo pre-counseling regarding the benefits and limitations of the noninvasive zygosity assessment. Chorionicity of twin pregnancies were identified by prenatal ultrasound at 7-8 gestational weeks. Maternal peripheral blood samples were collected between 17 and 26 weeks of gestation into EDTA-anticoagulated blood tubes before invasive prenatal procedures. And 1.5 mL of the husband's blood was collected. All samples were confirmed by invasive diagnosis and short tandem repeat (STR) typing. This study protocol was approved by the institutional review board of Shanghai First Maternity and Infant Hospital (GKLW2019-72 and GKLW2019-52).

**Workflow**

This study presents a single-blind retrospective investigation methodology focusing on zygosity assessment, and fetal fraction estimation in 55 twin pregnancies (Figure S1). Cell-free DNA was extracted from blood samples, followed by multiplex PCR enrichment targeting 1035 single nucleotide polymorphism (SNP) regions. Sequencing of PCR products was conducted on an Ion Proton sequencer, and subsequent data underwent quality control. Linkage Disequilibrium information-based noninvasive zygosity (LDNZ) assay determines zygosity via two binomial distributions, and the individual fetal fraction in the DZ twins was calculated using fetus-specific SNPs. The accuracy of the detection methods was evaluated using invasive diagnostic short tandem repeat (STR) results.

**SNP select and Primer design**

We utilized the East Asian reference panel from the 1000 Genomes Project Phase 3 (CHB + CHS + CDX + JPT + KHV, EAS_N = 504) as a sample pool for SNP sites selection. All SNP sites were required to meet the following criteria: (1) EAS minor allele frequency (MAF) > 0.4; (2) minimum inter distance >120 base pairs; (3) LD-blocks containing two main haplotypes (each frequency≥ 0.4). LD-blocks were selected by Plink software (v1.07) with default parameters for linkage disequilibrium thresholds (--blocks-strong-lowci 0.70 and --blocks-strong-highci 0.98) (Figure 1 A).

Primer3 software was employed to design primers targeting the selected SNPs, with a melting temperature (Tm) range of 57°C to 63°C and an average amplicon length of 75 base pairs. Thermodynamic calculations were conducted to avoid primer dimer formation. LD-blocks containing fewer than 5 SNPs were excluded. For LD-blocks containing 5 or more SNP sites meeting primer design criteria, only the top 5 SNP of MAF were retained. LD-blocks of chromosomes 1-22 comprising 1035 SNPs were obtained.

**Library Preparation and Target Sequencing**

Library construction was based on multiplex PCR amplification followed by amplicon sequencing, involving two-round PCR reactions. In the first round of PCR, amplification was performed simultaneously for 1035 target SNPs. Maternal cell-free DNA (cfDNA) and genomic DNA (gDNA) extracted from maternal and paternal samples, with cfDNA 5 ng and gDNA 10 ng, were utilized for library preparation. PCR amplification was conducted with 15 cycles (see Table S1-S4), followed by magnetic bead purification. Purified libraries were subjected to a second round of PCR amplification using primers containing barcoded sequences for 16 cycles. Amplification products were purified using magnetic beads and quantified using Qubit. After the normalization of libraries from multiple samples, they were pooled and sequenced on the Ion Proton platform using P1 chips, each capable of accommodating up to 48 samples. Sequencing data were aligned to the reference genome (hg19) using the TMAP software.

**Determination of Paternity, Zygosity and Fetal Fraction**

To assess the allele frequencies of 1035 SNPs, SNPs were classified into 3 categories based on the mother and alleged father genotypes, as described in Figure 1D. Category 1 SNPs represent homozygous genotypes for either the reference or alternative alleles in both parents. Category 2 SNPs represent a heterozygous genotype in the father and a homozygous genotype in the mother. Category 3 SNPs represent a homozygous genotype for the father's reference allele and the mother's alternative allele, or vice versa (Figure 1B).

Each block was treated as a unit. If more than 4 SNPs within a block belonged to the same category, the block was included in the subsequent LDNZ analysis. After removing outliers, each block's block mean and block coverage were calculated. Block mean (BM) refers to the mean frequency dosage change of SNP loci. And block coverage (BC) refers to the mean sequencing depth of SNP loci. The BM for Category 1 can be used to calculate the background value (*bg*), while the BM for Category 3 can be used to calculate the total fetal fraction (*ff_Total_*). The calculations for the *bg* and *ff_Total_* for twin pregnancies are as follows:

$\frac{{ff}_{Total}}{2}=\frac{1}{n}\left( \sum_{i=1}^{n} {BM(i)}_{\mathrm{Category}3} \right)$ (1)

$bg=\frac{1}{n}\left( \sum_{i=1}^{n} {BM(i)}_{\mathrm{Category}1} \right)$ (2)

Using *bg* and *ff_Total_* as the probability values and BC as the number of experimental simulations, two binomial distributions—X_bg_ and X_Total_ are generated. The probability of a block falling within X_bg_ and X_Total_ is calculated. The likelihood of the block's BM is in X_bg_ (P_bg_) and X_Total_ (P_total_) is calculated using the following formulas:

$X_{bg}\sim B\left( {BC,ff}_{bg} \right)$ (3)

$X_{total}\sim B\left( BC,\frac{{ff}_{Total}}{2} \right)$ (4)

The X_bg_ and X_Total_ of Category 2 are performed for zygosity assessment. If a Category 2 block meets the conditions of P_bg_ ≤ 0.0008 and P_total_ ≤ 0.0005, it indicates that the twins inherited different paternal haplotypes. These blocks are defined as informative blocks (Table 1). The sample would be identified as MZ twins if the total number of informative blocks is 0. The sample would be identified as DZ twins if the total number of informative blocks is ≥ 3. The result is inconclusive if the total number of informative blocks falls between 1 and 2. BM of informative blocks was classified into 2 groups by the K-means clustering algorithm to calculate individual fetal fractions in the DZ twins (See additional Material). The fetal fraction is defined as *ff_1_* for the lower fetal fraction and *ff_2_* for the higher fetal fraction.

**Results**

**Zygosity Assessment based on LDNZ**

For Category 2 locus where the mother is homozygous and father is heterozygosity, twins different genomes would cause additional block coverage expect for the ${ff}_{bg}$ and $\frac{{ff}_{total}}{2}$. Zygosity assessment was performed on the 55 twin pregnancies, revealing 28 dizygotic twins and 27 monozygotic twins (Table 1). Following the experimental methodology, analysis of the 207 target blocks in parents revealed 10 to 36 informative blocks in 28 DZ twins. In contrast, the remaining 27 MZ twins showed no informative blocks in the LDNZ analysis. A comparison of invasive STR zygosity typing results from the 55 twin pairs revealed 100% consistency (55/55) with the LDNZ results. Take M042 and M051 families for example (Figure 2). In the M042 twin pregnancy, none of Category 2 blocks met the criteria of P_0_ ≤ 0.05 and P_Total_ ≤ 0.05. Hence, M042 twins were identified as the MZ twin (Figure 2A). For M051 twin pregnancy, 33 informative blocks were observed, so M051 twins were identified as the DZ twin (Figure 2B).

The total fetal fraction for MZ twins ranged from 8.70% to 25.78%, with an average fetal fraction of 16.83% (Figure 2C). DZ twins' total fetal fraction ranged from 7.62% to 23.08%, with an average fetal fraction of 14.74% (Figure 2C). Furthermore, individual fetal fraction determination was completed for all DC twins (Figure 2D). *ff_1_* ranged from 2.48% to 9.17%, with an average *ff_1_* of 5.81%. *ff_2_* ranged from 4.15% to 13.34%, with an average *ff_2_* of 8.39%, which is 1.44 times greater than *ff_1_*.

**Zygosity Assessment based on SNP**

Applying classical SNP-based zygosity assessment, using individual SNP loci as a unit, revealed that 35 cases were DZ twins and 20 were MZ twins. In the LDNZ method, the cutoff value is set at 3 blocks, each containing 5 SNP loci. Therefore, for the classical SNP-based method, the cutoff value for the informative loci is 15. Between 17 and 160 informative SNPs were identified in the 35 DZ twins, while only 1 to14 informative SNPs were detected in the remaining 20 MZ twins. A comparison of the results with those of STR zygosity testing of 55 twin pairs revealed a detection accuracy of 87.27% (48/55). M024, M029, M035, M044, M046, M047, and M048, which were determined to be MZ twins by STR, were misclassified as DZ twins, with the number of informative SNPs being 26, 20, 27, 23, 17, 18, and 28.

The histogram and ROC (Receiver Operating Characteristic Curve) analysis were performed to compare the number of informative loci obtained by the two methods (Figure 3). The number of informative blocks identified by the LDNZ method exhibited a noticeable increase in DZ twins (Figure 3A). In contrast, the number of informative SNPs identified by the classical SNP-based method showed a continuous distribution without distinct cutoff points (Figure 3C). ROC analysis was used to evaluate the predictive accuracy of zygosity assessment methods at best cutoff thresholds. For the LDNZ method, when the cutoff for informative blocks was set at 1, 2, or 3 (Figure 3B), both sensitivity and specificity were 100%. On the contrary, for the classical SNP-based method (Figure 3D), at the best cutoff of 28 SNPs, specificity was 100%, and sensitivity was 93%. In the classical SNP-based analysis, regardless of the cutoff value set, the AUC never reached 1.

**Paternity Determination**

The X_bg_ and X_Total_ of Category 1 and 3 are performed for paternity determination. The principle of paternity determination is based on Mendelian inheritance laws. For the biological father, all the Category 3 blocks show P_0_ ≥ 0.05, and Category 1 blocks fulfill the condition of P_total_ ≥ 0.05. If more than three Category 1 or Category 3 blocks do not meet these criteria, a non-biological father or two non-biological fathers are identified as the biological fathers of the twin fetuses. Among 55 twin pregnancies, paternity was successfully identified in 55 twins, except for M009 twins with a non-biological father. In the M009 twins, 16 blocks of Category 3 and Category 1 were observed with P ≤ 0.05, including 3 Category 3 blocks and 13 Category 1 blocks. On the contrary, all the Category 3 and Category 1 blocks of the remaining 54 twin pregnancies show P＞0.05, confirming the alleged father (AF) is the biological father of both fetuses. Conventional STR typing was performed to verify the paternity and zygosity of both fetuses with the Goldeneye^®^ DNA ID 25A system. The STR results calculated PT and CPI following the Chinese national standards recommended for paternity testing (GB/T 37223-2018) to determine paternity.
